# Association of fat-to-muscle mass ratio with physical activity and dietary protein, carbohydrate, sodium, and fiber intake in a cross-sectional study

**DOI:** 10.1038/s41598-024-61289-8

**Published:** 2024-05-09

**Authors:** Shu Nishikori, Satoshi Fujita

**Affiliations:** 1 Faculty of Sport and Health Science, Ritsumeikan University, 1-1-1 Nojihigashi, Kusatsu, Japan; 2 Frontier Research Center, POLA Chemical Industries, Inc., 560 Kashio-cho, Totsuka-ku, Yokohama, Japan

**Keywords:** Health care, Risk factors

## Abstract

Higher fat-to-muscle mass ratio (FMR) is reported to be a risk factor for various diseases, including type 2 diabetes and cardiovascular diseases, and mortality. Although this association suggests that reducing FMR may help to prevent certain diseases and mortality, the relationship between FMR and lifestyle factors is unclear. Therefore, we performed a cross-sectional study with the aim to elucidate this relationship. This cross-sectional study included 1518 healthy Japanese adults aged 30 to 64 years. We measured FMR in the whole body, arms, legs, and trunk and assessed various lifestyle factors. Then, we performed forced entry multiple regression analyses for FMR with the following variables: sex, age, physical activity, dietary intake, sleep quality, cigarette smoking, stress levels, and body mass index. As a result, whole-body and regional FMRs were correlated with female sex (β = 0.71); age (β = 0.06); physical activity (β = − 0.07); dietary intake of protein (β = − 0.12), carbohydrate (β = 0.04), sodium (β = 0.13), and fiber (β = − 0.16); and body mass index (β = 0.70). The results suggest that in the Japanese middle-aged population, low FMR is associated with certain lifestyle factors, i.e. higher physical activity and a diet with higher protein and fiber and lower carbohydrate and sodium, independent of age, sex, and body mass index.

## Introduction

Aging- and lifestyle-related diseases such as cancer, type 2 diabetes, and cardiovascular diseases can decrease quality of life and eventually require nursing care and result in mortality. In aging societies, prevention of these diseases is becoming more important to maximize the length of healthy life.

Body mass index (BMI) is a common body composition parameter used as a risk indicator for certain diseases and mortality^1–4^. However, its use is a topic of ongoing debate^5–9^ because the body is composed mainly of fat and muscle, which are highly variable even among individuals with the same BMI. In other words, BMI is based on body weight without considering the distribution of fat and muscle.

Previous prospective cohort studies indicated that the fat mass index (fat mass divided by height square [kg/m^2^]) and muscle mass index (muscle mass divided by height square [kg/m^2^]) are positively and negatively associated, respectively, with all-cause mortality^10,11^, and other studies suggested that fat and muscle mass are more appropriate predictors of mortality than BMI^12,13^. Therefore, the ratio of fat mass to muscle mass, referred to as the fat-to-muscle mass ratio (FMR), is thought to be more useful than BMI. In fact, FMR was reported to be a predictor for type 2 diabetes^14^ and mortality^15^ independent of BMI. In addition, FMR was found to be associated with various clinical outcomes or markers such as cancer^16,17^, cardiometabolic risk factors^18,19^, hyperuricemia^20^, coronary artery disease^21^, liver stiffness^22^, insulin resistance and metabolic syndromes^23^.

The above findings suggest that FMR is an important risk factor for aging- and lifestyle-related diseases and that reducing FMR may help to prevent such diseases and mortality. However, the best approach for controlling FMR is unclear because no direct relationship has been reported between FMR and lifestyle factors. Therefore, to elucidate the relationship between FMR and lifestyle factors such as physical activity, dietary intake, sleep quality, cigarette smoking, and stress levels, we performed a cross-sectional study with a total of 1518 healthy Japanese adults aged 30 to 64 years and performed multiple regression analyses adjusted for age, sex, and BMI.

## Results

### Study participants

A total of 1518 healthy Japanese adults (50.9% men) aged 30 to 64 years (48.2 ± 10.0 years) participated in the study. The mean BMI was 22.8 ± 3.0 kg/m^2^.

### Correlation between FMR and BMI

Pearson correlation analyses were performed to confirm the correlation between body composition parameters, such as the various FMRs, and BMI. The results are shown in Extended Data Table 1. Whole-body and regional FMRs were highly correlated with each other (*r* = 0.94–0.99), but each FMR was only moderately correlated with BMI (*r* = 0.32–0.58).

### Sex differences in FMR and lifestyle factors

Participant characteristics, including body composition and lifestyle factors, and the results of comparative analyses between men and women are shown in Table 1. Sex differences were found in FMRs, BMI, physical activity, dietary intake of energy, protein, carbohydrate, vitamin, mineral, and alcohol, cigarette smoking, and stress levels.

**Table 1:** Participant characteristics.

	All (*n* = 1518)	Men (*n* = 773)	Women (*n* = 745)	*p*-value (men vs women)
Age, y	48.2 ± 10.0	48.4 ± 10.4	48.0 ± 9.6	0.42
Body composition				
Whole-body FMR	0.380 ± 0.148	0.31 ± 0.113	0.452 ± 0.146	< 0.0001
Arm FMR	0.490 ± 0.259	0.335 ± 0.161	0.651 ± 0.243	< 0.0001
Leg FMR	0.365 ± 0.139	0.284 ± 0.09	0.449 ± 0.131	< 0.0001
Trunk FMR	0.398 ± 0.158	0.334 ± 0.132	0.464 ± 0.155	< 0.0001
Body mass index, kg/m^2^	22.8 ± 3.0	23.7 ± 3.0	21.9 ± 2.7	< 0.0001
Physical activity (PA)				
Total PA, mets/day	298.4 ± 420.1	324.2 ± 440.6	271.6 ± 396.2	< 0.05
Vigorous PA, mets/day	100.5 ± 259.2	125.9 ± 292.5	74.2 ± 216.4	< 0.0001
Moderate PA, mets/day	53.5 ± 159.0	54.3 ± 148.5	52.6 ± 169.3	0.83
Walking PA, mets/day	144.4 ± 210.2	143.9 ± 189.3	144.8 ± 230.0	0.93
Dietary intake				
Energy, kcal/day	1635.5 ± 524.6	1729.6 ± 543.5	1537.8 ± 485.7	< 0.0001
Protein, g/day	60.7 ± 21.1	62.4 ± 22.1	58.9 ± 20.0	< 0.01
Animal protein, g/day	33.8 ± 15.1	34.6 ± 15.9	33.0 ± 14.1	< 0.05
Vegetable protein, g/day	26.9 ± 9.3	27.8 ± 9.6	25.9 ± 8.9	< 0.0001
Fat, g/day	50.9 ± 18.2	51.2 ± 18.5	50.5 ± 18.0	0.45
Animal fat, g/day	23.1 ± 9.8	23.4 ± 10.3	22.8 ± 9.3	0.28
Vegetable fat, g/day	27.7 ± 10.7	27.8 ± 10.4	27.7 ± 11.1	0.78
Carbohydrate, g/day	212.9 ± 78.2	226.6 ± 80.7	198.8 ± 73.0	< 0.0001
Vitamin, mg/day	140.3 ± 65.7	137.3 ± 65.3	143.3 ± 66.0	< 0.01
Mineral, g/day	16.1 ± 5.1	16.7 ± 5.3	15.5 ± 4.9	< 0.0001
Fiber, g/day	10.4 ± 4.5	10.2 ± 4.5	10.5 ± 4.4	0.22
Water, g/day	1651.7 ± 603.8	1755.9 ± 646.9	1543.5 ± 534.8	< 0.0001
Alcohol, g/day	8.9 ± 16.0	12.4 ± 19.8	5.3 ± 9.4	< 0.0001
Sleep quality				
PSQI global score	4.89 ± 2.54	4.79 ± 2.57	4.99 ± 2.52	0.13
Subjective sleep quality	1.22 ± 0.63	1.18 ± 0.62	1.26 ± 0.64	< 0.01
Sleep latency	0.79 ± 0.84	0.74 ± 0.81	0.84 ± 0.87	< 0.05
Sleep duration	1.20 ± 0.80	1.22 ± 0.81	1.18 ± 0.80	0.33
Habitual sleep efficiency	0.12 ± 0.42	0.11 ± 0.40	0.14 ± 0.43	0.25
Sleep disturbances	0.85 ± 0.48	0.82 ± 0.48	0.88 ± 0.48	< 0.05
Use of sleep medication	0.08 ± 0.42	0.10 ± 0.46	0.07 ± 0.36	0.14
Daytime dysfunction	0.62 ± 0.99	0.62 ± 1.00	0.62 ± 0.98	0.99
Cigarette smoking habit				
Cigarette pack-year	4.27 ± 8.96	6.64 ± 10.92	1.82 ± 5.30	< 0.0001
Stress				
PHRF-SCL total score	8.37 ± 8.02	6.56 ± 7.07	10.24 ± 8.51	< 0.0001
Anxiety/uncertainty	0.83 ± 1.32	0.57 ± 1.06	1.10 ± 1.49	< 0.0001
Tiredness/physical responses	3.61 ± 2.93	2.79 ± 2.53	4.46 ± 3.07	< 0.0001
Autonomic symptons	1.56 ± 2.51	1.27 ± 2.31	1.87 ± 2.67	< 0.0001
Depression/feeling of insufficiency	2.37 ± 2.79	1.94 ± 2.58	2.81 ± 2.93	< 0.0001

Values are the means ± standard deviations (SDs). Results in men and women were compared with 2-sided independent *t* tests.

*FMR* fat-to-muscle mass ratio, *PSQI* Pittsburgh sleep quality index, *PHRF*-*SCL* public health research foundation-stress check list.

Whole-body and regional FMRs were higher in women than men, and the ratios of FMR in women to men were as follows: whole body, 1.46 (0.45 in women vs 0.31 in men); arms, 1.94 (0.65 vs 0.34); legs, 1.58 (0.45 vs 0.28); and trunk, 1.39 (0.46 vs 0.33). In contrast, BMI was lower in women, with a ratio of FMR in women to men of 0.92 (21.9 vs 23.7).

Although the amount of total and vigorous physical activity was higher in men than women, the amount of moderate exercise and walking was not different between the sexes.

Regarding dietary factors, intake of energy, protein, carbohydrate, mineral, water, and alcohol was higher in men than women, whereas intake of vitamin was higher in women. Dietary intake of fat and fiber was not different between the sexes.

Although the PSQI global score, i.e. the total score for sleep quality, was not different between men and women, the scores for subjective sleep quality, sleep latency, and sleep disturbances were higher in women than men, indicating that women have more difficulties in those sleep components than men.

Regarding cigarette smoking, cigarette pack-years were higher in men than women.

In terms of stress levels, PHRF-SCL total and the scores for anxiety/uncertainty, tiredness/physical responses, autonomic symptoms, and depression/feeling of insufficiency were higher in women than men.

### Correlation between age and other variables

To confirm the correlation between age and other variables, we performed comparison analyses stratified by 10-year age groups from the 30 s to 60 s (Extended Data Table 2).

A tendency was found for whole-body and regional FMRs and BMI to be positively correlated with age. Regarding lifestyle factors, dietary intake apart from carbohydrate and cigarette smoking increased with age, whereas stress levels decreased specifically in participants in their 60 s.

### Multiple regression model for FMR with lifestyle factors adjusted for sex and age (model 1)

To elucidate which lifestyle factors were correlated with FMR, forced entry multiple regression analyses were performed for whole-body and regional FMRs with lifestyle factors adjusted for sex and age (model 1). The results for whole-body, arm, leg, and trunk FMRs are shown in Table 2 and Extended Data Tables 3, 4 and 5, respectively; almost the same trend was found for all FMRs. In model 1, female sex, age, dietary intake of carbohydrate, vitamin and mineral, PSQI global score, and cigarette pack-years were positively correlated with whole-body FMR, whereas total physical activity and dietary intake of protein and fiber were negatively correlated with it. The results stratified by sex showed a similar trend.

**Table 2:** Multiple regression models for whole-body fat-to-muscle mass ratio.

Variate	Model	All (*n* = 1518)			Men (*n* = 773)			Women (*n* = 745)		
		β	*t*	*p*-value	β	*t*	*p*-value	β	*t*	*p*-value
Female sex	1	0.506	20.2	< 0.0001	–	–	–	–	–	–
	2	0.703	42.8	< 0.0001	–	–	–	–	–	–
Age, y	1	0.113	4.9	< 0.0001	0.103	2.8	< 0.01	0.152	4.0	< 0.0001
	2	0.058	3.9	< 0.001	0.031	1.3	0.20	0.098	4.3	< 0.0001
Total PA, mets/day	1	− 0.082	− 3.7	< 0.001	− 0.140	− 4.0	< 0.0001	− 0.057	− 1.6	0.12
	2	− 0.072	− 5.1	< 0.0001	− 0.108	− 4.9	< 0.0001	− 0.070	− 3.2	< 0.01
Protein, g/day	1	− 0.195	− 2.6	< 0.05	− 0.178	− 1.5	0.13	− 0.285	− 2.2	< 0.05
	2	− 0.113	− 2.3	< 0.05	− 0.126	− 1.7	0.09	− 0.139	− 1.8	0.07
Fat, g/day	1	0.010	0.2	0.82	− 0.011	− 0.2	0.88	0.025	0.3	0.73
	2	− 0.017	− 0.6	0.55	− 0.031	− 0.7	0.50	− 0.017	− 0.4	0.71
Carbohydrate, g/day	1	0.091	3.0	< 0.01	0.086	1.9	0.06	0.116	2.2	< 0.05
	2	0.056	2.9	< 0.01	0.054	1.8	0.07	0.060	1.9	0.06
Vitamin, mg/day	1	0.135	2.6	< 0.01	0.171	2.3	< 0.05	0.155	1.7	0.09
	2	0.053	1.6	0.10	0.054	1.1	0.3	0.097	1.8	0.08
Mineral, g/day	1	0.258	3.2	< 0.01	0.307	2.5	< 0.05	0.304	2.2	< 0.05
	2	0.131	2.5	< 0.05	0.158	2.0	< 0.05	0.162	1.9	0.05
Fiber, g/day	1	− 0.337	− 6.0	< 0.0001	− 0.424	− 5.0	< 0.0001	− 0.372	− 3.7	< 0.001
	2	− 0.171	− 4.7	< 0.0001	− 0.192	− 3.5	< 0.001	− 0.236	− 3.9	< 0.0001
Alcohol, g/day	1	− 0.035	− 1.5	0.13	− 0.060	− 1.7	0.10	− 0.006	− 0.2	0.88
	2	− 0.002	− 0.1	0.92	− 0.006	− 0.3	0.78	0.006	0.3	0.78
PSQI global score	1	0.052	2.0	< 0.05	0.056	1.3	0.18	0.066	1.5	0.13
	2	0.023	1.4	0.17	0.014	0.5	0.59	0.037	1.4	0.15
Cigarette pack − year	1	0.067	2.9	< 0.01	0.108	2.9	< 0.01	0.063	1.7	0.08
	2	0.015	1.0	0.30	0.041	1.7	0.08	0.030	1.4	0.16
PHRF − SCL total score	1	0.049	1.8	0.07	0.043	1.0	0.30	0.059	1.4	0.18
	2	0.004	0.2	0.82	0.022	0.8	0.40	− 0.017	− 0.7	0.51
Body mass index, kg/m^2^	1	–	–	–	–	–	–	–	–	–
	2	0.698	47.3	< 0.0001	0.753	33.4	< 0.0001	0.783	36.1	< 0.0001

Forced entry multiple regression analysis was performed with 2 models: model 1, not included body mass index; model 2, included body mass index.

*FMR* fat-to-muscle mass ratio, *β* standardized coefficient, *PA* physical activity, *PSQI* Pittsburgh sleep quality index, *PHRF*-*SCL* public health research foundation-stress check list.

### Multiple regression model for FMR with lifestyle factors adjusted for sex, age, and BMI (model 2)

To confirm whether the lifestyle factors identified in model 1 were correlated with FMR independent of BMI, a multiple regression analysis was performed with BMI as an additional adjustment variable (model 2). In this model, dietary intake of vitamin, PSQI global score, and cigarette pack-years were not correlated with whole-body FMR, but female sex, age, total physical activity and dietary intake of carbohydrate, mineral, protein and fiber were significantly correlated with it, as was BMI. The results stratified by sex showed a similar trend.

### Multiple regression model for the association of FMR with lifestyle factors and intake of each individual mineral

To explore which mineral was correlated with FMR, we performed a multiple regression analysis with lifestyle factors and intake of the individual mineral rather than sum of mineral intakes. In both models 1 and 2, sodium intake was positively correlated with FMR (Extended Data Table 6). Therefore, we performed multiple regression analysis with lifestyle factors and sodium intake instead of sum of mineral intakes (Table 3).

**Table 3:** Multiple regression models for whole-body fat-to-muscle mass ratio with sodium as the mineral.

Variate	Model	All (*n* = 1518)			Men (*n* = 773)			Women (*n* = 745)		
		β	*t*	*p* − value	β	*t*	*p* − value	β	*t*	*p* − value
Female sex	1	0.521	20.8	< 0.0001	–	–	–	–	–	–
	2	0.710	43.2	< 0.0001	–	–	–	–	–	–
Age, y	1	0.113	4.9	< 0.0001	0.104	2.8	< 0.01	0.151	4.0	< 0.0001
	2	0.058	3.9	< 0.0001	0.031	1.3	0.19	0.098	4.3	< 0.0001
Total PA, mets/day	1	− 0.082	− 3.7	< 0.001	− 0.137	− 4.0	< 0.0001	− 0.058	− 1.6	0.11
	2	− 0.071	− 5.1	< 0.0001	− 0.106	− 4.8	< 0.0001	− 0.071	− 3.2	< 0.01
Protein, g/day	1	− 0.196	− 3.1	< 0.01	− 0.155	− 1.6	0.12	− 0.306	− 2.9	< 0.01
	2	− 0.115	− 2.9	< 0.01	− 0.115	− 1.8	0.07	− 0.149	− 2.4	< 0.05
Fat, g/day	1	0.005	0.1	0.91	− 0.019	− 0.3	0.80	0.021	0.3	0.78
	2	− 0.020	− 0.7	0.49	− 0.035	− 0.8	0.45	− 0.019	− 0.4	0.67
Carbohydrate, g/day	1	0.069	2.3	< 0.05	0.064	1.4	0.17	0.083	1.6	0.12
	2	0.044	2.3	< 0.05	0.042	1.4	0.15	0.043	1.4	0.18
Vitamin, mg/day	1	0.196	3.9	< 0.0001	0.244	3.3	< 0.01	0.220	2.5	< 0.05
	2	0.085	2.7	< 0.01	0.092	1.9	0.05	0.132	2.4	< 0.05
Sodium, g/day	1	0.241	5.4	< 0.0001	0.254	3.7	< 0.001	0.300	4.1	< 0.0001
	2	0.125	4.4	< 0.0001	0.132	3.0	< 0.01	0.157	3.5	< 0.001
Fiber, g/day	1	− 0.323	− 6.0	< 0.0001	− 0.410	− 5.1	< 0.0001	− 0.341	− 3.6	< 0.001
	2	− 0.164	− 4.8	< 0.0001	− 0.185	− 3.6	< 0.001	− 0.220	− 3.8	< 0.001
Alcohol, g/day	1	− 0.033	− 1.5	0.15	− 0.055	− 1.6	0.12	− 0.008	− 0.2	0.83
	2	− 0.001	− 0.1	0.96	− 0.004	− 0.2	0.87	0.005	0.2	0.82
PSQI global score	1	0.052	2.0	< 0.05	0.056	1.3	0.18	0.066	1.5	0.13
	2	0.023	1.4	0.17	0.014	0.5	0.59	0.038	1.5	0.15
Cigarette pack − year	1	0.064	2.8	< 0.01	0.104	2.8	< 0.01	0.065	1.8	0.07
	2	0.014	1.0	0.34	0.039	1.7	0.10	0.032	1.5	0.14
PHRF − SCL total score	1	0.049	1.8	0.07	0.043	1.0	0.31	0.058	1.4	0.18
	2	0.004	0.2	0.82	0.022	0.8	0.41	− 0.017	− 0.7	0.51
Body mass index, kg/m^2^	1	–	–	–	–	–	–	–	–	–
	2	0.695	47.2	< 0.0001	0.750	33.3	< 0.0001	0.778	36.0	< 0.0001

*FMR* fat-to-muscle mass ratio, *β* standardized coefficient, *PA* physical activity, *PSQI* Pittsburgh sleep quality index, *PHRF*-*SCL* public health research foundation-stress check list.

Forced entry multiple regression analysis was performed with 2 models: model 1, not included body mass index; model 2, included body mass index.

## Discussion

In this study, a multiple regression analysis adjusted for sex and age showed that FMR is associated with physical activity, dietary intake of protein, carbohydrate, vitamin, sodium, and fiber, sleep quality, and cigarette smoking. In a multiple regression analysis with additional adjustment for BMI, we found that FMR was associated with physical activity and dietary intake of protein, carbohydrate, sodium, and fiber. To our knowledge, this is the first study to show a direct correlation between FMR and lifestyle factors.

Generally, women are known to have higher fat mass and lower muscle mass than men in many populations^24–27^, including the Japanese^28,29^. These findings indicate that FMR, i.e. fat mass divided by muscle mass, is higher in women than men, as was found in this study (Table 1). For regional FMRs, the ratio of the FMR in women to men, in descending order, was 1.94 (0.651 in women vs 0.335 in men) for the arms, 1.58 (0.449 vs 0.284) for the legs, and 1.39 (0.464 vs 0.334) for the trunk. The ratio is lowest for the trunk because it includes visceral fat, which is more likely to be present in men than women^30–32^.

With aging, fat mass increases and muscle mass decreases, regardless of sex and population^24,26,28,29^, indicating that FMR increases with age. This study also found a trend of increasing FMR with age (Extended Data Table 2). However, no increase in FMR was found in women in their 60 s compared with women in younger age groups, i.e. the 30 s, 40 s, and 50 s. One reason for this finding might be that the women in their 60 s in this study had higher dietary protein and fiber intake, which contributed to a lower FMR.

Systematic reviews and meta-analyses have reported that exercise, especially aerobic exercise, decreases body weight, fat mass, and visceral fat mass^33^. Asian individuals with higher physical activity as a lifestyle habit have higher total muscle mass and lower fat mass in their limbs^34^. Systematic reviews and meta-analyses also reported that resistance training reduces body fat mass in healthy adults and individuals with overweight and obesity^35,36^. These findings indicate that physical activity is related to FMR. The present study found a significant correlation between physical activity and FMR in the multiple regression models, even after adjustment for sex, age, and BMI (Table 2).

A systematic review and meta-analysis showed a dose–response relationship between protein intake and muscle mass increase^37^. An increase in protein intake is reported to result in additional gains in muscle mass induced by resistance training^38^. These findings suggest that higher dietary protein intake is related to higher muscle mass, which contributes to a lower FMR, and our study also found a correlation between higher dietary protein intake and lower FMR in the multiple regression models, even after adjustment for sex, age, and BMI (Table 2).

A meta-analysis of randomized controlled studies suggested that a low-carbohydrate diet decreases fat mass in individuals with obesity^39^. The same effect was found in a study on individuals with type 2 diabetes^40^. These findings suggest that lower dietary carbohydrate intake is associated with lower FMR, and our study also found a positive correlation between dietary carbohydrate intake and FMR.

Our results showed that overall dietary vitamin intake was positively correlated with FMR after adjustment for sex and age (model 1 in Table 2). The multiple regression analysis with 12 individual vitamins identified vitamin C as a factor that was positively correlated with FMR (data not shown). In contrast to our study, previous studies found that a lower level of circulating vitamin C was correlated with higher BMI and fat mass^41,42^ and lower skeletal muscle mass^43,44^. On the other hand, higher vitamin C intake, extracted by principal component analysis, was related to higher BMI and body fat and correlated with sugar intake (*r* = 0.726)^45^. Therefore, we hypothesize that higher dietary vitamin C intake co-occurring with higher sugar intake increases fat mass and explains the positive correlation with FMR.

In our study, overall dietary mineral intake was found to positively correlate with FMR, even after adjustment for sex, age, and BMI, and a separate multiple regression analysis of the individual mineral identified sodium intake as positively correlated with FMR. This finding supports previous cross-sectional analyses that found positive correlations between sodium intake and body fat and BMI^46–49^, and identified higher sodium intake as a risk factor for obesity independent of potential confounders such as sex, age, and energy intake^50^. Furthermore, studies in several Asian populations reported that high salt intake was related to muscle strength and physical performance^51–53^. Although we have insufficient data to determine the mechanism behind the relationship between FMR and sodium intake, after adjusting for various confounders, studies found that dietary sodium intake is correlated with higher intake of sugar-sweetened beverages^48,54^, and systematic reviews and meta-analyses showed that consumption of sugar-sweetened beverages is a risk factor for weight gain and obesity^55–57^. Additionally, a study in an animal model found that high salt intake activates the aldose reductase-fructokinase pathway in the liver and hypothalamus, leading to endogenous fructose production and the development of leptin resistance and hyperphagia, which are known to cause obesity, insulin resistance, and fatty liver^58,59^.

Increasing fiber intake is reported to decrease weight and fat mass, independent of potential confounders such as physical activity and fat intake^60^. Furthermore, a review suggested that increasing fiber intake reduces energy intake and weight, which may help to decrease high levels of obesity in populations^61^. Our finding of a negative correlation between dietary fiber intake and FMR is in agreement with these findings.

Sleep is essential and important for a healthy life. It is also relevant for body composition because it regulates fat, muscle, and bone mass through the endocrine system and promotes the secretion of growth hormones^62^. Previous studies showed that lower sleep quality and shorter sleep duration are correlated with higher fat mass and lower muscle and bone mass^63–65^. We also confirmed in our study that higher sleep quality contributed to lower FMR.

Cigarette smoking is a risk factor for type 2 diabetes. As concerns the mechanism, research has suggested that nicotine negatively affects insulin sensitivity in skeletal muscle and increases lipolysis^66,67^. A history of smoking was found to be correlated with fat mass and waist circumference^68,69^. Cigarette smoking also correlated with FMR in our study.

The correlation analyses between FMR and BMI showed significant but modest correlations (*r* = 0.32–0.58, Extended Data Table 1). These results suggest that FMR, which is based on the distribution of fat and muscle, and BMI, which is based on body weight, have different physiological implications. In addition, they support previous studies that showed that FMR is a predictor for type 2 diabetes^14^ and mortality^15^ independent of BMI and our finding that FMR is correlated with several lifestyle factors independent of BMI. These findings may suggest that FMR is a more useful indicator of a healthy lifestyle and the risk of lifestyle-related diseases than BMI.

The FMR cutoff value for metabolic syndrome in a Chinese population was suggested to be 0.34 for men and 0.55 for women^70^, and the FMR cutoff for impaired insulin sensitivity in patients with treatment-naïve type 2 diabetes in a Japanese population was found to be 2.75 (0.36 when estimated as FMR) for men and 1.65 (0.61 when estimated as FMR) for women^71^. It is reasonable that the mean FMR values in men (0.310) and women (0.452) in our study were lower than the above cutoff values because the participants in our study were healthy individuals.

There are some limitations to this study. First, the relationship between FMR and lifestyle factors is valid for this sample population but may not be applicable to other populations with different lifestyles. Westerners have lower FMR than Asians, probably because they have higher muscle mass^24^, and sex-specific reference values for FMR may also be different between Asian populations, e.g. the mean values of FMR in Japanese men and women in this study were higher and lower, respectively, than those found in a Chinese population^72^. Therefore, further studies are needed to confirm that the relationships are valid in other cohorts. Second, the study was cross-sectional, so we are unable to conclude whether modification of these lifestyle factors actually attenuates FMR and subsequently contributes to reduction of disease and mortality-related risk factors.

In conclusion, FMR, which has been proposed as a useful indicator for various aging- and lifestyle-related diseases and mortality, was found to show a positive correlation with dietary intake of carbohydrate and sodium and a negative correlation with physical activity and dietary intake of protein and fiber when analyses were adjusted for sex, age, and BMI. This finding suggests that exercise and a diet with higher intake of protein and fiber and lower intake of carbohydrate and sodium may help to achieve and maintain a lower FMR and prevent aging- and lifestyle-related diseases. To confirm this hypothesis, future lifestyle intervention and longitudinal studies are needed.

## Methods

### Study design and participants

This cross-sectional study included 1,518 healthy Japanese aged 30 to 64 years living in or near Tokyo, including Kanagawa, Chiba, and Saitama prefectures. All measurements were performed in December 2020, and participants were prohibited from changing their lifestyle habits and introducing new behaviors, such as dietary restriction, excessive exercise, and drinking alcohol, for one month before the measurement day.

The study complied with all relevant ethical regulations. It was approved by the ethics committees of TES Holdings Co., Ltd., Taito-ku, Tokyo, Japan (HR-2022-POM4), and POLA Chemical Industries, Inc., Yokohama, Japan (2020-F-141) and was conducted in accordance with the Declaration of Helsinki. All participants were informed about the experimental procedures and possible risks, and all of them provided written informed consent.

### Measurement of body composition

Body composition was measured by the direct segmental multi-frequency bio-electrical impedance analysis method with a body composition analyzer (Inbody770, Inbody Japan, Koto-ku, Tokyo), which measures impedance (Z) at 6 frequencies (1, 5, 50, 250, 500, and 1000 kHz), reactance (Xc) at 3 frequencies (5, 50 and 250 kHz), and the phase angle (θ) at a frequency of 50 kHz. We measured the whole body and 5 regional sites (right and left arm, right and left leg, and trunk) and used the analyzer to calculate body composition parameters such as weight, fat mass, and muscle mass at all measurement sites. We selected the Inbody770 for body composition analysis because it calculates body composition parameters only from impedance values and does not make any statistical adjustments for variables such as sex and age.

FMR was defined as fat mass divided by muscle mass measured by a body composition analyzer (Inbody770) and calculated with the formula used in previous studies^14,15,20^. The various FMRs were calculated as follows: whole body (fat mass [total]/muscle mass [total]); arms (fat mass [right arm + left arm]/muscle mass [right arm + left arm]), legs (fat mass [right leg + left leg]/muscle mass [right leg + left leg]), and trunk (fat mass [trunk]/muscle mass [trunk]). BMI was defined as weight divided by height squared (kg/m^2^).

### Assessment of lifestyle factors

Lifestyle factors such as physical activity, dietary intake, sleep quality, cigarette smoking, and physical and psychological stress levels that were considered to be potentially related to fat or muscle mass or both were evaluated with validated questionnaires.

Physical activity was evaluated with the international physical activity questionnaire (IPAQ)^73,74^, which asks about vigorous and moderate exercise and walking. Total physical activity, i.e. the sum of scores for vigorous and moderate exercise and walking, was used for multiple regression analysis.

Dietary intake was evaluated with the brief-type self-administered diet history questionnaire (BDHQ)^75,76^, which assesses the dietary intake of about 100 nutrients, such as animal- and vegetable-derived protein and fat, carbohydrate, vitamin and mineral, soluble and insoluble fiber, and alcohol. The dietary intake of each nutrient was calculated by referring to the Food Composition Database developed by the Japanese Ministry of Education, Culture, Sports, Science and Technology. The database includes 13 vitamins (vitamins A, C, D, E, K, B1, B2, B6, and B12, niacin, folic acid, pantothenic acid, and biotin) and 13 minerals (sodium, potassium, calcium, magnesium, phosphorus, iron, zinc, copper, manganese, iodine, selenium, chromium, and molybdenum); however, the vitamin biotin and the 4 minerals iodine, selenium, chromium, and molybdenum were excluded from the BDHQ results in this study. Thus, the data on vitamins and minerals in the Tables indicates the dietary intake of 12 vitamins (without biotin) and 9 minerals (without iodine, selenium, chromium, and molybdenum). Then, dietary intake of energy, protein, fat, carbohydrate, vitamin, minerals (sodium, potassium, calcium, magnesium, phosphorus, iron, zinc, copper, and manganese), fiber, and alcohol was included in multiple regression analysis to clarify correlations with FMR.

Sleep quality was assessed with the Pittsburgh sleep quality index (PSQI)^77–79^, which evaluates various aspects of sleep, such as sleep latency and duration, and sleep disturbances. The PSQI global score, a sum of all sleep quality scores, was used for multiple regression analysis.

Cigarette smoking was evaluated as cigarette pack-years^80^, calculated as the mean number of cigarettes smoked divided by 20 (the number of cigarettes usually included in a pack) and multiplied by the number of smoking years. Cigarette pack-years were included in the multiple regression analysis.

Stress levels were estimated with the Public Health Research Foundation-Stress Check List (PHRF-SCL)^81^, which evaluates various aspects of stress, such as anxiety/uncertainty, tiredness/physical responses, autonomic symptoms, and depression/feeling of insufficiency. The PHRF-SCL score, i.e., the sum of all stress scores, was used for multiple regression analysis.

### Statistical analysis

Differences in characteristics between men and women were analyzed with 2-sided independent *t* tests (Table 1). Multiple regression analyses were performed by the forced entry and least square methods with adjustment for sex and age (model 1) and age, sex, and BMI (model 2). Correlations were analyzed by Pearson correlation analysis.

All statistical analyses were performed with JMP version 16 (SAS Institute, Cary, NC, USA). Probability values of less than 0.05 (p < 0.05) were considered significant.

### Supplementary Information

Supplementary Information.

## Data Availability

The data that support the findings of this study are available from the corresponding author on reasonable request.
