# Supplementary material for: Association of fat-to-muscle mass ratio with physical activity and dietary protein, carbohydrate, sodium, and fiber intake in a cross-sectional study
Source: Sci Rep. 2024 May 9;14:10631. doi: 10.1038/s41598-024-61289-8 (PMC11082163; doi:10.1038/s41598-024-61289-8)
Supplement: Supplementary file 1 — Supplementary Information. [file 41598_2024_61289_MOESM1_ESM.pdf]

Association of fat-to-muscle mass ratio with physical activity and dietary protein, carbohydrate, sodium, and fiber intake in a cross-sectional study

Shu Nishikori[1,2] and Satoshi Fujita[1]

[1] Faculty of Sport and Health Science, Ritsumeikan University, 1-1-1 Nojihigashi, Kusatsu, Japan

[2] Frontier Research Center, POLA Chemical Industries, Inc., 560 Kashio-cho, Totsuka-ku, Yokohama, Japan

Corresponding author:
*Satoshi Fujita[1]
Tel: +81-077-561-5229, E-mail: safujita@fc.ritsumei.ac.jp

Author:
Shu Nishikori[1,2]
Tel: +81-090-2734-7983, E-mail: s-nishikori@pola.co.jp

Extended Data Table 1. Correlation coefficient of whole-body fat-to-muscle mass ratio, regional fat-to-muscle mass ratios, and body mass index

Variable	Whole-body FMR		Arm FMR		Leg FMR		Trunk FMR		Body mass index	
	r	*p* value	*r*	*p* value	*r*	*p* value	*r*	*p* value	*r*	*p* value
Whole-body FMR			–	–	–	–	–	–	–	–
Arm FMR	0.97	<0.0001			–	–	–	–	–	–
Leg FMR	0.98	<0.0001	0.98	<0.0001			–	–	–	–
Trunk FMR	0.99	<0.0001	0.94	<0.0001	0.95	<0.0001			–	–
Body mass index	0.51	<0.0001	0.32	<0.0001	0.38	<0.0001	0.58	<0.0001		

FMR, fat-to-muscle mass ratio

r shows the Pearson correlation coeffieient between variables.

Extended Data Table 2. Correlations between age and other variables

Variable	Sex	Age group: 30s All (*n* = 378) Men (*n* = 198) Women (*n* = 180)		40s All (*n* = 396) Men (*n* = 195) Women (*n* = 201)		50s All (*n* = 477) Men (*n* = 219) Women (*n* = 258)		60s All (*n* = 267) Men (*n* = 161) Women (*n* = 106)		*p* value					
		Mean	S.D.	Mean	S.D.	Mean	S.D.	Mean	S.D.	30s *vs* 40s	30s *vs* 50s	30s *vs* 60s	40s *vs* 50s	40s *vs* 60s	50s *vs* 60s
Body composition															
Whole-body FMR	All	0.356	0.008	0.372	0.007	0.403	0.007	0.382	0.009	0.47	<0.0001	0.12	<0.01	0.80	0.24
	Men	0.287	0.008	0.310	0.008	0.320	0.008	0.325	0.009	0.19	<0.05	<0.05	0.81	0.57	0.96
	Women	0.432	0.011	0.431	0.010	0.474	0.009	0.468	0.014	1.00	<0.05	0.17	<0.01	0.14	0.99
Arm FMR	All	0.457	0.013	0.473	0.013	0.533	0.012	0.486	0.016	0.83	<0.001	0.51	<0.01	0.92	0.08
	Men	0.307	0.011	0.332	0.011	0.349	0.011	0.356	0.013	0.40	<0.05	<0.05	0.71	0.49	0.97
	Women	0.623	0.018	0.609	0.017	0.689	0.015	0.682	0.023	0.95	<0.05	0.18	<0.01	0.06	0.99
Leg FMR	All	0.347	0.007	0.356	0.007	0.387	0.006	0.363	0.008	0.80	<0.001	0.43	<0.01	0.89	0.11
	Men	0.266	0.006	0.283	0.006	0.291	0.006	0.294	0.007	0.24	<0.05	<0.05	0.79	0.65	0.99
	Women	0.435	0.010	0.426	0.009	0.468	0.008	0.468	0.013	0.90	<0.05	0.16	<0.01	<0.05	1.00
Trunk FMR	All	0.371	0.008	0.390	0.008	0.422	0.007	0.404	0.010	0.33	<0.0001	<0.05	<0.05	0.66	0.47
	Men	0.306	0.009	0.334	0.009	0.344	0.009	0.354	0.010	0.15	<0.05	<0.01	0.85	0.50	0.91
	Women	0.442	0.012	0.444	0.011	0.487	0.010	0.481	0.015	1.00	<0.05	0.17	<0.05	0.19	0.99
Body mass index, kg/m^2	All	22.4	0.15	22.8	0.15	23.0	0.14	23.2	0.18	0.14	<0.05	<0.01	0.94	0.34	0.63
	Men	23.1	0.21	23.8	0.21	23.9	0.20	24.1	0.23	0.08	<0.05	<0.01	0.99	0.78	0.89
	Women	21.6	0.20	21.9	0.19	22.2	0.17	21.9	0.26	0.70	0.13	0.79	0.71	1.00	0.83
Physical activity (PA)															
Total PA, mets/day	All	297.9	21.6	294.2	21.1	298.8	19.3	304.2	25.7	1.00	1.00	1.00	1.00	0.99	1.00
	Men	336.1	31.3	331.3	31.6	289.2	29.8	348.4	34.7	1.00	0.70	0.99	0.77	0.98	0.57
	Women	255.9	29.5	258.3	27.9	307.1	24.7	237.1	38.5	1.00	0.54	0.98	0.56	0.97	0.42
Vigorous PA, mets/day	All	107.8	13.3	105.0	13.0	97.3	11.9	89.3	15.9	1.00	0.94	0.81	0.97	0.87	0.98
	Men	154.6	20.8	117.3	21.0	123.0	19.8	105.2	23.1	0.59	0.69	0.38	1.00	0.98	0.94
	Women	56.3	16.1	93.0	15.3	75.5	13.5	65.3	21.0	0.35	0.80	0.99	0.83	0.71	0.98
Moderate PA, mets/day	All	51.3	8.2	50.8	8.0	52.8	7.3	61.6	9.7	1.00	1.00	0.85	1.00	0.83	0.89
	Men	49.2	10.5	65.1	10.6	33.3	10.0	76.2	11.7	0.71	0.69	0.32	0.13	0.90	<0.05
	Women	53.7	12.6	36.9	11.9	69.4	10.5	39.4	16.4	0.77	0.77	0.90	0.17	1.00	0.42
Walking, mets/day	All	138.8	10.8	138.5	10.6	148.7	9.6	153.3	12.9	1.00	0.90	0.82	0.89	0.81	0.99
	Men	132.3	13.4	148.9	13.5	132.9	12.8	167.0	14.9	0.82	1.00	0.31	0.82	0.81	0.30
	Women	145.9	17.1	128.3	16.2	162.2	14.3	132.4	22.3	0.88	0.89	0.96	0.40	1.00	0.68
Dietary intake															
Energy, kcal/day	All	1516.2	26.6	1596.0	26.0	1678.2	23.7	1786.5	31.6	0.14	<0.0001	<0.0001	0.09	<0.0001	<0.05
	Men	1688.1	38.5	1682.1	38.8	1726.5	36.6	1842.3	42.6	1.00	0.89	<0.05	0.84	<0.05	0.17
	Women	1327.1	34.8	1512.5	33.0	1637.3	29.1	1701.8	45.4	<0.001	<0.0001	<0.0001	<0.05	<0.01	0.63
Protein, g/day	All	56.1	1.1	59.1	1.0	61.8	1.0	67.6	1.3	0.20	<0.001	<0.0001	0.21	<0.0001	<0.01
	Men	60.5	1.6	61.4	1.6	60.7	1.5	68.2	1.7	0.98	1.00	<0.01	0.99	<0.05	<0.01
	Women	51.3	1.4	56.9	1.4	62.7	1.2	66.5	1.9	<0.05	<0.0001	<0.0001	<0.01	<0.001	0.32
Animal protein, g/day	All	31.9	0.8	32.8	0.8	33.8	0.7	38.2	0.9	0.83	0.22	<0.0001	0.72	<0.0001	<0.001
	Men	34.1	1.1	34.2	1.1	32.5	1.1	38.8	1.2	1.00	0.73	<0.05	0.71	<0.05	<0.001
	Women	29.4	1.0	31.5	1.0	35.0	0.9	37.3	1.3	0.48	<0.001	<0.0001	<0.05	<0.01	0.45
Vegetable protein, g/day	All	24.2	0.5	26.3	0.5	28.0	0.4	29.4	0.6	<0.05	<0.0001	<0.0001	<0.05	<0.001	0.20
	Men	26.4	0.7	27.2	0.7	28.2	0.6	29.5	0.8	0.83	0.20	<0.05	0.69	0.12	0.60
	Women	21.9	0.6	25.4	0.6	27.8	0.5	29.2	0.8	<0.001	<0.0001	<0.0001	<0.05	<0.01	0.46
Fat, g/day	All	47.0	0.9	50.3	0.9	51.6	0.8	55.9	1.1	0.06	<0.01	<0.0001	0.69	<0.001	<0.05
	Men	49.2	1.3	51.3	1.3	50.0	1.2	55.3	1.4	0.69	0.98	<0.01	0.89	0.16	<0.05
	Women	44.6	1.3	49.3	1.2	53.0	1.1	56.7	1.7	<0.05	<0.0001	<0.0001	0.11	<0.01	0.28
Animal fat, g/day	All	22.1	0.5	22.9	0.5	22.8	0.4	25.4	0.6	0.71	0.73	<0.001	1.00	<0.01	<0.01
	Men	23.4	0.7	23.6	0.7	22.0	0.7	25.0	0.8	1.00	0.49	0.44	0.36	0.58	<0.05
	Women	20.8	0.7	22.2	0.6	23.6	0.6	25.9	0.9	0.44	<0.01	<0.0001	0.36	<0.01	0.14
Vegetable fat, g/day	All	24.9	0.5	27.4	0.5	28.8	0.5	30.5	0.6	<0.01	<0.0001	<0.0001	0.21	<0.01	0.15
	Men	25.8	0.7	27.6	0.7	28.0	0.7	30.3	0.8	0.31	0.14	<0.001	0.98	0.08	0.14
	Women	23.8	0.8	27.2	0.8	29.5	0.7	30.8	1.0	<0.05	<0.0001	<0.0001	0.10	<0.05	0.71
Carbohydrate, g/day	All	197.6	4.0	208.4	3.9	220.9	3.6	227.0	4.7	0.21	<0.0001	<0.0001	0.08	<0.05	0.73
	Men	225.3	5.7	220.5	5.8	229.3	5.5	231.8	6.4	0.94	0.96	0.87	0.69	0.56	0.99
	Women	167.1	5.2	196.7	5.0	213.8	4.4	219.8	6.8	<0.001	<0.0001	<0.0001	<0.05	<0.05	0.88
Vitamin, mg/day	All	122.0	3.3	129.7	3.2	148.9	2.9	166.2	3.9	0.34	<0.0001	<0.0001	<0.0001	<0.0001	<0.01
	Men	125.6	4.6	127.2	4.6	139.2	4.3	161.3	5.0	1.00	0.13	<0.0001	0.22	<0.0001	<0.01
	Women	118.1	4.7	132.2	4.5	157.2	3.9	173.7	6.1	0.13	<0.0001	<0.0001	<0.001	<0.0001	0.11
Mineral, g/day	All	14.8	0.3	15.6	0.3	16.5	0.2	18.0	0.3	0.11	<0.0001	<0.0001	<0.05	<0.0001	<0.001
	Men	15.9	0.4	16.2	0.4	16.6	0.4	18.3	0.4	0.92	0.60	<0.001	0.93	<0.01	<0.01
	Women	13.5	0.3	14.9	0.3	16.5	0.3	17.4	0.5	<0.05	<0.0001	<0.0001	<0.01	<0.0001	0.27
Fiber, g/day	All	9.2	0.2	9.8	0.2	10.9	0.2	11.9	0.3	0.18	<0.0001	<0.0001	<0.01	<0.0001	<0.01
	Men	9.5	0.3	9.7	0.3	10.3	0.3	11.6	0.3	0.99	0.24	<0.0001	0.39	<0.001	<0.05
	Women	8.8	0.3	10.0	0.3	11.3	0.3	12.4	0.4	<0.05	<0.0001	<0.0001	<0.01	<0.0001	0.13
Water, g/day	All	1494.0	30.4	1582.4	29.7	1733.6	27.1	1831.5	36.2	0.16	<0.0001	<0.0001	<0.01	<0.0001	0.13
	Men	1683.2	45.6	1653.3	45.9	1817.6	43.3	1886.0	50.6	0.97	0.14	<0.05	<0.05	<0.01	0.73
	Women	1286.0	38.0	1513.7	36.0	1662.2	31.7	1748.7	49.5	<0.0001	<0.0001	<0.0001	<0.05	<0.001	0.46
Alcohol, g/day	All	8.1	0.8	7.4	0.8	9.0	0.7	12.3	1.0	0.93	0.84	<0.01	0.45	<0.001	<0.05
	Men	10.6	1.4	9.3	1.4	13.1	1.3	17.3	1.5	0.93	0.55	<0.01	0.21	<0.001	0.17
	Women	5.3	0.7	5.4	0.7	5.4	0.6	4.7	0.9	1.00	1.00	0.95	1.00	0.91	0.90
Sleep quality															
PSQI global score	All	4.88	0.13	4.89	0.13	4.91	0.12	4.86	0.16	1.00	1.00	1.00	1.00	1.00	0.99
	Men	4.63	0.18	4.94	0.18	4.77	0.17	4.84	0.20	0.61	0.94	0.86	0.90	0.98	0.99
	Women	5.16	0.19	4.85	0.18	5.03	0.16	4.89	0.24	0.63	0.96	0.82	0.86	1.00	0.96
Subjective sleep quality	All	1.23	0.03	1.20	0.03	1.26	0.03	1.16	0.04	0.94	0.94	0.56	0.63	0.86	0.23
	Men	1.13	0.04	1.21	0.04	1.21	0.04	1.16	0.05	0.64	0.57	0.97	1.00	0.91	0.88
	Women	1.34	0.05	1.20	0.04	1.29	0.04	1.17	0.06	0.16	0.89	0.13	0.43	0.97	0.32
Sleep latency	All	0.87	0.04	0.80	0.04	0.71	0.04	0.78	0.05	0.65	<0.05	0.50	0.39	0.98	0.73
	Men	0.79	0.06	0.77	0.06	0.68	0.05	0.70	0.06	1.00	0.56	0.70	0.67	0.80	1.00
	Women	0.97	0.06	0.83	0.06	0.74	0.05	0.91	0.08	0.43	<0.05	0.94	0.66	0.89	0.33
Sleep duration	All	1.10	0.04	1.22	0.04	1.31	0.04	1.12	0.05	0.16	<0.001	0.98	0.28	0.45	<0.01
	Men	1.13	0.06	1.29	0.06	1.29	0.05	1.16	0.06	0.22	0.20	0.99	1.00	0.46	0.44
	Women	1.06	0.06	1.15	0.06	1.34	0.05	1.07	0.08	0.70	<0.01	1.00	0.06	0.82	<0.05
Habitual sleep efficiency	All	0.13	0.02	0.14	0.02	0.09	0.02	0.14	0.03	1.00	0.50	0.99	0.35	1.00	0.39
	Men	0.11	0.03	0.13	0.03	0.06	0.03	0.16	0.03	0.97	0.62	0.72	0.36	0.92	0.12
	Women	0.16	0.03	0.15	0.03	0.12	0.03	0.12	0.04	1.00	0.79	0.93	0.85	0.96	1.00
Sleep disturbances	All	0.80	0.02	0.84	0.02	0.86	0.02	0.91	0.03	0.76	0.28	<0.05	0.87	0.28	0.65
	Men	0.74	0.03	0.83	0.03	0.83	0.03	0.91	0.04	0.27	0.24	<0.01	1.00	0.32	0.31
	Women	0.88	0.04	0.85	0.03	0.90	0.03	0.90	0.05	0.95	0.98	0.99	0.75	0.86	1.00
Use of sleep medication	All	0.06	0.02	0.08	0.02	0.08	0.02	0.13	0.03	0.79	1.00	0.14	0.84	0.54	0.44
	Men	0.06	0.03	0.12	0.03	0.11	0.03	0.12	0.04	0.54	0.70	0.58	0.99	1.00	0.99
	Women	0.06	0.03	0.05	0.03	0.06	0.02	0.14	0.03	1.00	1.00	0.21	0.99	0.15	0.19
Daytime dysfunction	All	0.69	0.05	0.61	0.05	0.60	0.05	0.61	0.06	0.71	0.56	0.81	1.00	1.00	0.99
	Men	0.67	0.07	0.61	0.07	0.59	0.07	0.63	0.08	0.91	0.86	0.98	1.00	0.99	0.98
	Women	0.70	0.07	0.61	0.07	0.60	0.06	0.58	0.10	0.82	0.70	0.78	1.00	1.00	1.00
Cigarette smoking															
Cigarette pack-years	All	1.46	0.45	3.64	0.44	5.04	0.40	7.80	0.53	<0.01	<0.0001	<0.0001	0.09	<0.0001	<0.001
	Men	1.92	0.74	5.52	0.74	8.09	0.70	11.81	0.82	<0.01	<0.0001	<0.0001	0.06	<0.0001	<0.01
	Women	0.96	0.39	1.82	0.37	2.46	0.33	1.72	0.51	0.38	<0.05	0.64	0.58	1.00	0.62
Stress															
PHRF-SCL total score	All	9.28	0.41	8.44	0.40	8.58	0.37	6.59	0.49	0.46	0.58	<0.001	0.99	<0.05	<0.01
	Men	7.37	0.50	6.42	0.51	6.51	0.48	5.83	0.56	0.53	0.59	0.17	1.00	0.86	0.79
	Women	11.37	0.63	10.41	0.60	10.34	0.53	7.75	0.82	0.68	0.59	<0.01	1.00	<0.05	<0.05
Anxiety/uncertainty	All	0.92	0.07	0.85	0.07	0.77	0.06	0.79	0.08	0.89	0.36	0.64	0.80	0.94	1.00
	Men	0.55	0.08	0.52	0.08	0.55	0.07	0.67	0.08	0.99	1.00	0.71	0.99	0.53	0.68
	Women	1.32	0.11	1.17	0.11	0.95	0.09	0.97	0.14	0.77	0.06	0.23	0.41	0.69	1.00
Tiredness/physical responses	All	3.57	0.15	3.66	0.15	3.83	0.13	3.16	0.18	0.97	0.57	0.29	0.83	0.13	<0.05
	Men	2.86	0.18	2.71	0.18	2.79	0.17	2.80	0.20	0.94	0.99	1.00	0.99	0.99	1.00
	Women	4.36	0.23	4.59	0.22	4.72	0.19	3.72	0.30	0.88	0.62	0.31	0.97	0.08	<0.05
Autonomic symptons	All	2.05	0.13	1.48	0.13	1.55	0.11	1.03	0.15	<0.01	<0.05	<0.0001	0.97	0.10	<0.05
	Men	1.75	0.16	1.17	0.16	1.20	0.15	0.88	0.18	0.06	0.07	<0.01	1.00	0.64	0.54
	Women	2.39	0.20	1.78	0.19	1.84	0.17	1.25	0.26	0.11	0.15	<0.01	0.99	0.34	0.20
Depression/feeling of insufficiency	All	2.74	0.14	2.45	0.14	2.43	0.13	1.61	0.17	0.48	0.38	<0.0001	1.00	<0.001	<0.001
	Men	2.22	0.18	2.02	0.18	1.97	0.17	1.48	0.20	0.87	0.77	<0.05	1.00	0.20	0.25
	Women	3.31	0.22	2.87	0.20	2.82	0.18	1.82	0.28	0.46	0.31	<0.001	1.00	<0.05	<0.05

FMR, fat-to-muscle mass ratio; PSQI, Pittsburgh Sleep Quality Index; PHRF-SCL, Public Health Research Foundation-Stress Check List
Tukey-Kramer tests were performed to analyze correlations between age and other variables.

Extended Data Table 3. Multiple regression models for arm fat-to-muscle mass ratio

Variable	Model	All (*n* = 1518)			Men (*n* = 773)			Women (*n* = 745)		
		β	*t*	*p* value	β	*t*	*p* value	β	*t*	*p* value
Female sex	1	0.634	27.97	<0.0001	—	—	—	—	—	—
	2	0.784	44.11	<0.0001	—	—	—	—	—	—
Age, y	1	0.102	4.82	<0.0001	0.097	2.58	<0.05	0.155	4.07	<0.0001
	2	0.059	3.69	<0.001	0.032	1.17	0.24	0.110	3.85	<0.001
Total PA, mets/day	1	-0.076	-3.78	<0.001	-0.141	-4.07	<0.0001	-0.067	-1.84	0.07
	2	-0.067	-4.46	<0.0001	-0.113	-4.46	<0.0001	-0.078	-2.87	<0.01
Protein, g/day	1	-0.203	-2.95	<0.01	-0.211	-1.80	<0.01	-0.330	-2.55	<0.05
	2	-0.140	-2.68	<0.01	-0.165	-1.94	0.05	-0.209	-2.16	<0.05
Fat, g/day	1	0.013	0.31	0.76	0.004	0.05	0.96	0.018	0.25	0.81
	2	-0.008	-0.26	0.79	-0.014	-0.27	0.79	-0.017	-0.30	0.76
Carbohydrate, g/day	1	0.089	3.24	<0.01	0.089	1.94	0.05	0.131	2.49	<0.05
	2	0.061	2.96	<0.01	0.060	1.80	0.07	0.084	2.14	<0.05
Vitamin, mg/day	1	0.117	2.53	<0.05	0.167	2.20	<0.05	0.164	1.78	0.08
	2	0.054	1.55	0.12	0.062	1.12	0.26	0.116	1.68	0.09
Mineral, g/day	1	0.232	3.17	<0.01	0.285	2.31	<0.05	0.331	2.37	<0.05
	2	0.136	2.43	<0.05	0.152	1.69	0.09	0.212	2.04	<0.05
Fiber, g/day	1	-0.292	-5.70	<0.0001	-0.396	-4.65	<0.0001	-0.384	-3.85	<0.001
	2	-0.165	-4.23	<0.0001	-0.188	-3.02	<0.01	-0.271	-3.63	<0.001
Alcohol, g/day	1	-0.027	-1.28	0.20	-0.057	-1.59	0.11	0.006	0.16	0.87
	2	-0.001	-0.08	0.94	-0.009	-0.35	0.73	0.016	0.58	0.56
PSQI global score	1	0.052	2.17	<0.05	0.070	1.68	0.09	0.068	1.57	0.12
	2	0.029	1.62	0.11	0.033	1.09	0.28	0.044	1.37	0.17
Cigarette pack-years	1	0.043	2.03	<0.05	0.095	2.58	<0.05	0.045	1.24	0.22
	2	0.004	0.24	0.81	0.035	1.30	0.19	0.018	0.66	0.51
PHRF-SCL total score	1	0.030	1.23	0.22	0.034	0.80	0.42	0.037	0.84	0.40
	2	-0.004	-0.23	0.82	0.015	0.49	0.62	-0.027	-0.82	0.41
Body mass index, kg/m^2	1	—	—	—	—	—	—	—	—	—
	2	0.532	33.34	<0.0001	0.673	26.13	<0.0001	0.651	24.12	<.0001

FMR, fat-to-muscle mass ratio; β, standardized coefficient; PA, physical activity; PSQI, Pittsburgh Sleep Quality Index; PHRF-SCL, Public Health Research Foundation-Stress Check List

Forced entry multiple regression analysis was performed with 2 models: model 1, not included body mass index; model 2, included body mass index.

Extended Data Table 4. Multiple regression models for leg fat-to-muscle mass ratio

Variable	Model	All (n = 1518)			Men (n = 773)			Women (n = 745)		
		β	t	p value	β	t	p value	β	t	p value
Female sex	1	0.617	26.80	<0.0001	—	—	—	—	—	—
	2	0.786	47.82	<0.0001	—	—	—	—	—	—
Age, y	1	0.103	4.81	<0.0001	0.101	2.68	<0.01	0.152	3.99	<0.0001
	2	0.055	3.72	<0.001	0.032	1.24	0.21	0.101	4.03	<0.0001
Total PA, mets/day	1	-0.068	-3.34	<0.001	-0.137	-3.92	<0.0001	-0.048	-1.30	0.19
	2	-0.059	-4.19	<0.0001	-0.106	-4.42	<0.0001	-0.060	-2.52	<0.05
Protein, g/day	1	-0.170	-2.44	<0.05	-0.166	-1.41	0.16	-0.282	-2.17	<0.05
	2	-0.099	-2.06	<0.05	-0.117	-1.44	0.15	-0.143	-1.69	0.09
Fat, g/day	1	0.005	0.12	0.91	-0.013	-0.18	0.86	0.016	0.22	0.83
	2	-0.019	-0.65	0.52	-0.032	-0.63	0.53	-0.024	-0.49	0.63
Carbohydrate, g/day	1	0.075	2.72	<0.01	0.074	1.60	<0.05	0.105	1.98	<0.05
	2	0.045	2.33	<0.05	0.043	1.36	0.17	0.051	1.49	0.14
Vitamin, mg/day	1	0.120	2.56	<0.05	0.173	2.29	<0.05	0.152	1.64	0.10
	2	0.049	1.52	0.13	0.062	1.19	0.23	0.097	1.61	0.11
Mineral, g/day	1	0.219	2.94	<0.01	0.276	2.24	<0.05	0.301	2.15	<0.05
	2	0.110	2.14	<0.05	0.135	1.59	0.11	0.166	1.82	0.07
Fiber, g/day	1	-0.291	-5.60	<0.0001	-0.403	-4.72	<0.0001	-0.362	-3.61	<0.001
	2	-0.148	-4.10	<0.0001	-0.183	-3.10	<0.01	-0.232	-3.55	<0.001
Alcohol, g/day	1	-0.034	-1.62	0.11	-0.060	-1.68	0.09	-0.017	-0.47	0.64
	2	-0.006	-0.38	0.70	-0.010	-0.39	0.69	-0.006	-0.24	0.81
PSQI global score	1	0.056	2.30	<0.05	0.062	1.47	0.14	0.083	1.90	0.06
	2	0.031	1.83	0.07	0.023	0.78	0.43	0.055	1.95	0.05
Cigarette pack-years	1	0.048	2.22	<0.05	0.098	2.67	<0.01	0.048	1.32	0.19
	2	0.004	0.25	0.80	0.035	1.37	0.17	0.017	0.71	0.48
PHRF-SCL total score	1	0.035	1.41	0.16	0.038	0.90	0.37	0.041	0.94	0.35
	2	-0.004	-0.23	0.82	0.018	0.62	0.54	-0.032	-1.11	0.27
Body mass index, kg/m^2	1	—	—	—	—	—	—	—	—	—
	2	0.600	40.64	<0.0001	0.711	29.08	<0.0001	0.746	31.61	<0.0001

FMR, fat-to-muscle mass ratio; β, standardized coefficient; PA, physical activity; PSQI, Pittsburgh Sleep Quality Index; PHRF-SCL, Public Health Research Foundation-Stress Check List

Forced entry multiple regression analysis was performed with 2 models: model 1, not included body mass index; model 2, included body mass index.

Extended Data Table 5. Multiple regression models for trunk fat-to-muscle mass ratio

Variable	Model	All (n = 1518)			Men (n = 773)			Women (n = 745)		
		β	t	p value	β	t	p value	β	t	p value
Female sex	1	0.443	17.04	<0.0001	—	—	—	—	—	—
	2	0.656	41.55	<0.0001	—	—	—	—	—	—
Age, y	1	0.118	4.88	<0.0001	0.109	2.90	<0.01	0.149	3.92	<0.0001
	2	0.058	4.05	<0.0001	0.032	1.47	0.14	0.093	4.29	<0.0001
Total PA, mets/day	1	-0.085	-3.72	<0.001	-0.137	-3.93	<0.0001	-0.054	-1.49	0.14
	2	-0.074	-5.48	<0.0001	-0.103	-5.05	<0.0001	-0.068	-3.26	<0.01
Protein, g/day	1	-0.192	-2.45	<0.05	-0.159	-1.35	0.18	-0.283	-2.19	<0.05
	2	-0.103	-2.22	<0.05	-0.105	-1.53	0.13	-0.133	-1.80	0.07
Fat, g/day	1	0.015	0.33	0.74	0.001	0.01	0.99	0.027	0.36	0.72
	2	-0.014	-0.52	0.61	-0.020	-0.48	0.63	-0.016	-0.38	0.70
Carbohydrate, g/day	1	0.089	2.84	<0.01	0.079	1.71	0.09	0.113	2.14	<0.05
	2	0.050	2.72	<0.01	0.044	1.65	0.10	0.055	1.84	0.07
Vitamin, mg/day	1	0.144	2.71	<0.01	0.173	2.29	<0.05	0.152	1.65	0.10
	2	0.054	1.75	0.08	0.049	1.11	0.27	0.093	1.77	0.08
Mineral, g/day	1	0.268	3.19	<0.01	0.296	2.40	<0.05	0.313	2.24	<0.05
	2	0.130	2.63	<0.01	0.139	1.93	0.05	0.167	2.09	<0.05
Fiber, g/day	1	-0.356	-6.07	<0.0001	-0.428	-5.03	<0.0001	-0.373	-3.73	<0.001
	2	-0.175	-5.06	<0.0001	-0.182	-3.64	<0.001	-0.233	-4.08	<0.0001
Alcohol, g/day	1	-0.036	-1.49	0.14	-0.057	-1.60	0.11	-0.006	-0.16	0.87
	2	0.0005	0.03	0.97	-0.001	-0.04	0.97	0.006	0.29	0.77
PSQI global score	1	0.054	1.98	<0.05	0.051	1.21	0.23	0.070	1.61	0.11
	2	0.022	1.39	0.16	0.007	0.29	0.77	0.041	1.64	0.10
Cigarette pack-years	1	0.073	2.98	<0.01	0.105	2.85	<0.01	0.066	1.81	0.07
	2	0.017	1.17	0.24	0.034	1.57	0.12	0.032	1.56	0.12
PHRF-SCL total score	1	0.051	1.81	0.07	0.043	1.02	0.31	0.059	1.35	0.18
	2	0.002	0.10	0.92	0.021	0.84	0.40	-0.019	-0.77	0.44
Body mass index, kg/m^2	1	—	—	—	—	—	—	—	—	—
	2	0.757	53.41	<0.0001	0.794	38.35	<0.0001	0.804	38.97	<0.0001

FMR, fat-to-muscle mass ratio; β, standardized coefficient; PA, physical activity; PSQI, Pittsburgh Sleep Quality Index; PHRF-SCL, Public Health Research Foundation-Stress Check List

Forced entry multiple regression analysis was performed with 2 models: model 1, not included body mass index; model 2, included body mass index.

Extended Data Table 6. Multiple regression models for whole-body fat-to-muscle mass ratio with subdivided mineral intake

Variable	Model	All (*n* = 1518)			Men (*n* = 773)			Women (*n* = 745)		
		β	*t*	*p* value	β	*t*	*p* value	β	*t*	*p* value
Female sex	1	0.524	20.67	<0.0001	—	—	—	—	—	—
	2	0.706	42.41	<0.0001	—	—	—	—	—	—
Age, y	1	0.115	4.96	<0.0001	0.100	2.68	<0.01	0.158	4.15	<0.0001
	2	0.059	3.98	<0.0001	0.029	1.22	0.22	0.104	4.50	<0.0001
Total PA, mets/day	1	-0.077	-3.53	<0.001	-0.129	-3.72	<0.001	-0.057	-1.59	0.11
	2	-0.071	-5.08	<0.0001	-0.105	-4.77	<0.0001	-0.070	-3.23	<0.01
Protein, g/day	1	-0.129	-0.61	0.54	-0.103	-0.31	0.75	-0.020	-0.05	0.96
	2	-0.073	-0.53	0.59	-0.101	-0.48	0.63	0.076	0.34	0.74
Fat, g/day	1	0.002	0.03	0.97	-0.013	-0.15	0.88	0.027	0.33	0.74
	2	-0.030	-0.93	0.35	-0.013	-0.25	0.80	-0.043	-0.85	0.40
Carbohydrate, g/day	1	-0.133	-2.27	<0.05	-0.101	-1.12	0.26	-0.195	-1.93	0.05
	2	-0.019	-0.51	0.61	-0.008	-0.14	0.89	-0.034	-0.55	0.58
Vitamin, mg/day	1	0.236	2.75	<0.01	0.318	2.22	<0.05	0.237	1.73	0.08
	2	0.134	2.44	<0.05	0.118	1.29	0.20	0.193	2.32	<0.05
Mineral										
Sodium, mg/day	1	0.258	5.14	<0.0001	0.248	3.14	<0.01	0.329	4.08	<0.0001
	2	0.121	3.76	<0.001	0.106	2.09	<0.05	0.171	3.49	<0.001
Potassium, mg/day	1	-0.024	-0.15	0.88	-0.159	-0.58	0.56	0.154	0.57	0.57
	2	-0.074	-0.71	0.48	-0.047	-0.27	0.79	-0.025	-0.15	0.88
Calcium, mg/day	1	0.038	0.40	0.69	0.126	0.85	0.39	0.058	0.35	0.72
	2	0.010	0.17	0.87	-0.012	-0.12	0.90	0.134	1.33	0.18
Magnesium, mg/day	1	-0.373	-2.19	<0.05	-0.351	-1.28	0.20	-0.615	-2.12	<0.05
	2	-0.178	-1.63	0.10	-0.286	-1.64	0.10	-0.288	-1.64	0.10
Phosphorus, mg/day	1	-0.012	-0.04	0.96	-0.020	-0.05	0.96	-0.245	-0.53	0.60
	2	0.086	0.50	0.62	0.304	1.13	0.26	-0.295	-1.05	0.30
Iron, mg/day	1	-0.225	-2.19	<0.05	-0.087	-0.53	0.60	-0.378	-2.22	<0.05
	2	-0.106	-1.61	0.11	-0.108	-1.03	0.30	-0.090	-0.87	0.38
Zinc, mg/day	1	0.197	1.78	0.08	0.087	0.46	0.64	0.281	1.65	0.10
	2	0.057	0.81	0.42	-0.136	-1.13	0.26	0.170	1.64	0.10
Copper, mg/day	1	0.185	1.46	0.14	0.204	1.03	0.31	0.310	1.42	0.15
	2	0.034	0.42	0.68	0.117	0.92	0.36	0.086	0.65	0.51
Manganese, mg/day	1	0.122	2.90	<0.01	0.119	1.78	0.07	0.146	2.13	<0.05
	2	0.040	1.50	0.13	0.055	1.29	0.20	0.025	0.60	0.55
Fiber, g/day	1	-0.153	-1.87	0.06	-0.282	-2.24	<0.05	-0.119	-0.82	0.41
	2	-0.048	-0.92	0.36	-0.056	-0.69	0.49	-0.125	-1.43	0.15
Alcohol, g/day	1	0.024	0.87	0.39	0.016	0.35	0.72	0.035	0.87	0.39
	2	0.024	1.36	0.17	0.024	0.85	0.39	0.032	1.32	0.19
PSQI global score	1	0.049	1.87	0.06	0.052	1.25	0.21	0.063	1.47	0.14
	2	0.022	1.32	0.19	0.015	0.58	0.56	0.035	1.34	0.18
Cigarette pack-years	1	0.071	3.06	<0.01	0.110	2.99	<0.01	0.079	2.22	<0.05
	2	0.018	1.24	0.21	0.046	1.93	0.05	0.037	1.73	0.08
PHRF-SCL total score	1	0.052	1.96	<0.05	0.049	1.19	0.24	0.060	1.41	0.16
	2	0.006	0.33	0.74	0.023	0.88	0.38	-0.013	-0.52	0.61
Body mass index, kg/m^2	1	—	—	—	—	—	—	—	—	—
	2	0.690	46.60	<0.0001	0.749	33.05	<0.0001	0.770	35.35	<0.0001

FMR, fat-to-muscle mass ratio; β, standardized coefficient; PA, physical activity; PSQI, Pittsburgh Sleep Quality Index; PHRF-SCL, Public Health Research Foundation-Stress Check List

Forced entry multiple regression analysis was performed with 2 models: model 1, not included body mass index; model 2, included body mass index.
